# Hexa-μ_2_-acetato-κ^12^ *O*:*O*′-μ_3_-oxido-tris­[aqua­chromium(III)] nitrate acetic acid solvate

**DOI:** 10.1107/S1600536808029681

**Published:** 2008-09-24

**Authors:** Mohd Jamil Maah, Che Ibrahim Abdullah, Seng Neon Gan, Aishah Mohd Jelan, Seik Weng Ng

**Affiliations:** a Department of Chemistry, University of Malaya, 50603 Kuala Lumpur, Malaysia; b Centre for Foundation Studies in Science, University of Malaya, 50603 Kuala Lumpur, Malaysia

## Abstract

In the crystal structure of the title salt, [Cr_3_(C_2_H_3_O_2_)_6_O(H_2_O)_3_]NO_3_·CH_3_CO_2_H, the trinuclear [Cr_3_(CH_3_CO_2_)_6_O(H_2_O)_3_] cluster cation has an oxide O atom that is connected to three water-coordinated Cr^III^ atoms, the three metal atoms forming the points of an equilateral triangle. Each of the six acetate carboxyl­ate groups bridges a Cr–O–Cr fragment. The cluster cation inter­acts with the nitrate counter-ion and solvent mol­ecules through O—H⋯O hydrogen bonds, forming a three-dimensional hydrogen-bonded network.

## Related literature

For crystal structure reports of [Cr_3_(C_2_H_3_O_2_)_6_O(H_2_O)_3_]^+^ salts, see: Anson *et al.* (1997 ▶); Fujihara *et al.* (1998 ▶); Glowiak *et al.* (1996 ▶); Karu *et al.* (1993 ▶); Winpenny *et al.* (2005 ▶).
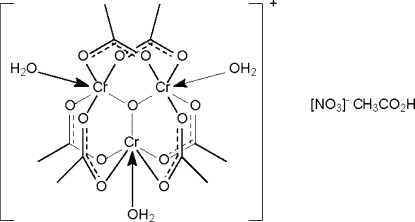


## Experimental

### 

#### Crystal data

[Cr_3_(C_2_H_3_O_2_)_6_O(H_2_O)_3_]NO_3_·C_2_H_4_O_2_
*M* *_r_* = 702.37
Monoclinic, 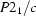

*a* = 11.7034 (1) Å
*b* = 14.5102 (2) Å
*c* = 15.0427 (2) Å
β = 91.532 (1)°
*V* = 2553.62 (5) Å^3^
*Z* = 4
Mo *K*α radiation
μ = 1.35 mm^−1^
*T* = 100 (2) K
0.20 × 0.10 × 0.05 mm

#### Data collection

Bruker SMART APEX diffractometer
Absorption correction: multi-scan (*SADABS*; Sheldrick, 1996 ▶) *T*_min_ = 0.774, *T*_max_ = 0.936
22819 measured reflections
5836 independent reflections
5092 reflections with *I* > 2σ(*I*)
*R*_int_ = 0.046

#### Refinement

*R*[*F* ^2^ > 2σ(*F* ^2^)] = 0.067
*wR*(*F* ^2^) = 0.224
*S* = 1.38
5836 reflections
359 parameters
H-atom parameters constrained
Δρ_max_ = 1.77 e Å^−3^
Δρ_min_ = −1.33 e Å^−3^

### 

Data collection: *APEX2* (Bruker, 2007 ▶); cell refinement: *SAINT* (Bruker, 2007 ▶); data reduction: *SAINT*; program(s) used to solve structure: *SHELXS97* (Sheldrick, 2008 ▶); program(s) used to refine structure: *SHELXL97* (Sheldrick, 2008 ▶); molecular graphics: *X-SEED* (Barbour, 2001 ▶); software used to prepare material for publication: *publCIF* (Westrip, 2008 ▶).

## Supplementary Material

Crystal structure: contains datablocks I, global. DOI: 10.1107/S1600536808029681/tk2306sup1.cif

Structure factors: contains datablocks I. DOI: 10.1107/S1600536808029681/tk2306Isup2.hkl

Additional supplementary materials:  crystallographic information; 3D view; checkCIF report

## Figures and Tables

**Table 1:** Hydrogen-bond geometry (Å, °)

*D*—H⋯*A*	*D*—H	H⋯*A*	*D*⋯*A*	*D*—H⋯*A*
O1w—H11⋯O16	0.84	1.96	2.769 (6)	162
O1w—H12⋯O12^i^	0.84	2.06	2.873 (5)	162
O2w—H21⋯O14	0.84	1.92	2.668 (6)	147
O2w—H22⋯O18^ii^	0.84	1.93	2.725 (6)	157
O3w—H31⋯O14^iii^	0.84	2.28	2.781 (5)	118
O3w—H32⋯O5^iii^	0.84	2.42	3.244 (6)	165
O15—H15⋯O18^iv^	0.84	1.81	2.624 (6)	163

Symmetry codes: (i) 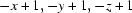; (ii) 
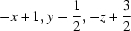
; (iii) 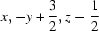; (iv) 
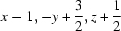
.

**Fig. 1.**
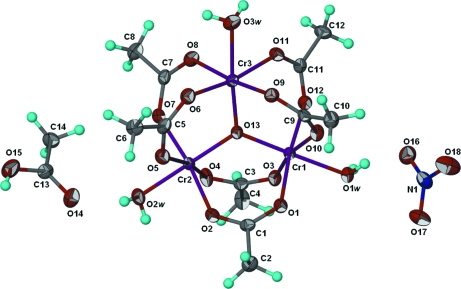
Thermal ellipsoid (Barbour, 2001) plot of [Cr3(C2H3O2)6O(H2O)3].[NO3].CH3CO2H at the 70% probability level. Hydrogen atoms are drawn as spheres of arbitrary radius.

## supplementary crystallographic information

### Experimental

Chromium(III) nitrate nonahydrate (10.06 g, 0.025 mol) was heated in acetic acid (24 ml, 0.42 mol) for 10 h. The solution was filtered and the acetic acid allowed to evaporate slowly. The dark-green crystals that separated were washed with chloroform and then dried at 433 K for 24 h. Yield: 70%.

### Refinement

The O- and C-bound H-atoms were placed in calculated positions with O—H = 0.84 Å and C—H 0.98 Å, and were included in the refinement in the riding model approximation, with *U*(H) set to 1.5 *U*(O, C). The final difference Fourier map had a large peak/deep hole at about 1 Å from the Cr3 atom.

### Crystal data

**Table:**

[Cr_3_(C_2_H_3_O_2_)_6_O(H_2_O)_3_]NO_3_·C_2_H_4_O_2_	*F*(000) = 1436
*M**_r_* = 702.37	*D*_x_ = 1.827 Mg m^−^^3^
Monoclinic, *P*2_1_/*c*	Mo *K*α radiation, λ = 0.71073 Å
Hall symbol: -P 2ybc	Cell parameters from 9927 reflections
*a* = 11.7034 (1) Å	θ = 2.2–28.2°
*b* = 14.5102 (2) Å	µ = 1.35 mm^−^^1^
*c* = 15.0427 (2) Å	*T* = 100 K
β = 91.532 (1)°	Chip, green
*V* = 2553.62 (5) Å^3^	0.20 × 0.10 × 0.05 mm
*Z* = 4	

### Data collection

**Table:**

Bruker SMART APEX diffractometer	5836 independent reflections
Radiation source: fine-focus sealed tube	5092 reflections with *I* > 2σ(*I*)
graphite	*R*_int_ = 0.046
ω scans	θ_max_ = 27.5°, θ_min_ = 2.0°
Absorption correction: multi-scan (SADABS; Sheldrick, 1996)	*h* = −15→15
*T*_min_ = 0.774, *T*_max_ = 0.936	*k* = −18→18
22819 measured reflections	*l* = −19→19

### Refinement

**Table:**

Refinement on *F*^2^	Primary atom site location: structure-invariant direct methods
Least-squares matrix: full	Secondary atom site location: difference Fourier map
*R*[*F*^2^ > 2σ(*F*^2^)] = 0.067	Hydrogen site location: inferred from neighbouring sites
*wR*(*F*^2^) = 0.224	H-atom parameters constrained
*S* = 1.38	*w* = 1/[σ^2^(*F*_o_^2^) + (0.1*P*)^2^ + 1*P*] where *P* = (*F*_o_^2^ + 2*F*_c_^2^)/3
5836 reflections	(Δ/σ)_max_ = 0.001
359 parameters	Δρ_max_ = 1.77 e Å^−^^3^
0 restraints	Δρ_min_ = −1.33 e Å^−^^3^

### Special details

**Table:**

Geometry. All e.s.d.'s (except the e.s.d. in the dihedral angle between two l.s. planes) are estimated using the full covariance matrix. The cell e.s.d.'s are taken into account individually in the estimation of e.s.d.'s in distances, angles and torsion angles; correlations between e.s.d.'s in cell parameters are only used when they are defined by crystal symmetry. An approximate (isotropic) treatment of cell e.s.d.'s is used for estimating e.s.d.'s involving l.s. planes.

### Fractional atomic coordinates and isotropic or equivalent isotropic displacement parameters (Å^2^)

**Table:**

	*x*	*y*	*z*	*U*_iso_*/*U*_eq_	
Cr1	0.40542 (6)	0.60336 (5)	0.62841 (5)	0.0131 (2)	
Cr2	0.17034 (6)	0.56625 (5)	0.73703 (5)	0.0135 (2)	
Cr3	0.18005 (6)	0.73269 (6)	0.58877 (5)	0.0140 (2)	
O1	0.4536 (3)	0.6151 (3)	0.7549 (2)	0.0198 (8)	
O2	0.2966 (3)	0.5724 (3)	0.8261 (3)	0.0205 (8)	
O3	0.3914 (3)	0.4689 (3)	0.6374 (3)	0.0184 (7)	
O4	0.2208 (3)	0.4442 (3)	0.6949 (3)	0.0198 (8)	
O5	0.1056 (3)	0.6751 (3)	0.7966 (3)	0.0195 (7)	
O6	0.0969 (3)	0.7819 (3)	0.6894 (3)	0.0195 (8)	
O7	0.0316 (3)	0.5543 (3)	0.6608 (3)	0.0189 (7)	
O8	0.0452 (3)	0.6588 (3)	0.5512 (3)	0.0192 (7)	
O9	0.3030 (3)	0.8221 (3)	0.6195 (2)	0.0174 (7)	
O10	0.4560 (3)	0.7309 (3)	0.6132 (3)	0.0193 (8)	
O11	0.2517 (3)	0.7039 (3)	0.4743 (2)	0.0176 (7)	
O12	0.3748 (3)	0.5871 (3)	0.4972 (2)	0.0172 (7)	
O13	0.2517 (3)	0.6341 (2)	0.6511 (2)	0.0147 (7)	
O14	−0.0709 (3)	0.5678 (3)	0.9306 (3)	0.0256 (9)	
O15	−0.2549 (4)	0.5997 (3)	0.9442 (3)	0.0299 (10)	
H15	−0.2433	0.6221	0.9951	0.045*	
O16	0.7058 (3)	0.7280 (3)	0.5673 (3)	0.0267 (9)	
O17	0.8176 (4)	0.7184 (3)	0.6842 (3)	0.0290 (9)	
O18	0.8263 (4)	0.8364 (3)	0.5964 (3)	0.0358 (11)	
O1W	0.5731 (3)	0.5752 (3)	0.6032 (2)	0.0172 (7)	
H11	0.6042	0.6224	0.5825	0.026*	
H12	0.5765	0.5321	0.5662	0.026*	
O2W	0.0843 (3)	0.4914 (3)	0.8268 (3)	0.0200 (8)	
H21	0.0592	0.5266	0.8661	0.030*	
H22	0.1285	0.4524	0.8505	0.030*	
O3W	0.1111 (3)	0.8387 (3)	0.5119 (3)	0.0222 (8)	
H31	0.0424	0.8465	0.5247	0.033*	
H32	0.1152	0.8250	0.4578	0.033*	
N1	0.7827 (4)	0.7597 (3)	0.6176 (3)	0.0214 (9)	
C1	0.3976 (4)	0.6011 (3)	0.8241 (3)	0.0161 (9)	
C2	0.4557 (5)	0.6207 (4)	0.9128 (4)	0.0213 (10)	
H2A	0.4404	0.6845	0.9302	0.032*	
H2B	0.5383	0.6116	0.9081	0.032*	
H2C	0.4260	0.5787	0.9577	0.032*	
C3	0.3122 (4)	0.4166 (4)	0.6615 (3)	0.0163 (9)	
C4	0.3268 (5)	0.3144 (4)	0.6485 (4)	0.0233 (11)	
H4A	0.3005	0.2816	0.7011	0.035*	
H4B	0.4077	0.3005	0.6398	0.035*	
H4C	0.2818	0.2946	0.5961	0.035*	
C5	0.0764 (4)	0.7537 (4)	0.7646 (4)	0.0175 (10)	
C6	0.0117 (5)	0.8165 (4)	0.8254 (4)	0.0213 (11)	
H6A	−0.0703	0.8035	0.8194	0.032*	
H6B	0.0259	0.8809	0.8092	0.032*	
H6C	0.0375	0.8060	0.8871	0.032*	
C7	−0.0024 (4)	0.5925 (4)	0.5887 (3)	0.0173 (10)	
C8	−0.1111 (4)	0.5551 (4)	0.5464 (4)	0.0217 (11)	
H8A	−0.1116	0.5672	0.4823	0.033*	
H8B	−0.1771	0.5854	0.5727	0.033*	
H8C	−0.1154	0.4886	0.5567	0.033*	
C9	0.4087 (4)	0.8085 (4)	0.6238 (3)	0.0163 (9)	
C10	0.4856 (5)	0.8895 (4)	0.6411 (4)	0.0214 (11)	
H10A	0.4417	0.9466	0.6336	0.032*	
H10B	0.5480	0.8887	0.5991	0.032*	
H10C	0.5172	0.8862	0.7020	0.032*	
C11	0.3182 (4)	0.6420 (3)	0.4472 (3)	0.0154 (9)	
C12	0.3291 (5)	0.6330 (4)	0.3480 (3)	0.0213 (10)	
H12A	0.2693	0.5918	0.3244	0.032*	
H12B	0.4044	0.6076	0.3349	0.032*	
H12C	0.3208	0.6939	0.3203	0.032*	
C13	−0.1699 (4)	0.5671 (4)	0.9000 (4)	0.0196 (10)	
C14	−0.2012 (5)	0.5280 (4)	0.8109 (4)	0.0220 (11)	
H14A	−0.1439	0.5463	0.7679	0.033*	
H14B	−0.2763	0.5515	0.7914	0.033*	
H14C	−0.2040	0.4606	0.8148	0.033*	

### Atomic displacement parameters (Å^2^)

**Table:**

	*U*^11^	*U*^22^	*U*^33^	*U*^12^	*U*^13^	*U*^23^
Cr1	0.0129 (4)	0.0149 (4)	0.0115 (4)	0.0003 (3)	0.0011 (3)	0.0001 (3)
Cr2	0.0135 (4)	0.0149 (4)	0.0123 (4)	0.0005 (3)	0.0018 (3)	−0.0001 (3)
Cr3	0.0134 (4)	0.0149 (4)	0.0136 (4)	0.0010 (3)	0.0001 (3)	−0.0013 (3)
O1	0.0163 (16)	0.029 (2)	0.0142 (17)	−0.0013 (14)	0.0007 (13)	0.0001 (15)
O2	0.0194 (18)	0.027 (2)	0.0154 (17)	−0.0023 (15)	0.0007 (14)	0.0032 (15)
O3	0.0155 (16)	0.0158 (17)	0.0241 (19)	0.0014 (13)	0.0044 (14)	0.0029 (14)
O4	0.0209 (18)	0.0149 (17)	0.0239 (19)	−0.0013 (14)	0.0088 (15)	−0.0018 (14)
O5	0.0207 (17)	0.0184 (18)	0.0194 (18)	0.0023 (14)	0.0033 (14)	−0.0001 (15)
O6	0.0187 (17)	0.0204 (18)	0.0196 (19)	0.0021 (14)	0.0020 (14)	−0.0027 (15)
O7	0.0152 (16)	0.0233 (19)	0.0182 (18)	−0.0021 (14)	0.0017 (13)	0.0031 (15)
O8	0.0177 (17)	0.0194 (18)	0.0205 (18)	0.0000 (14)	−0.0014 (14)	−0.0024 (15)
O9	0.0172 (17)	0.0167 (17)	0.0182 (17)	−0.0009 (13)	−0.0010 (13)	0.0004 (14)
O10	0.0164 (17)	0.0166 (17)	0.025 (2)	0.0004 (13)	0.0038 (14)	0.0010 (15)
O11	0.0204 (17)	0.0190 (18)	0.0136 (17)	0.0005 (14)	0.0010 (13)	0.0003 (14)
O12	0.0171 (17)	0.0208 (18)	0.0136 (16)	0.0012 (14)	0.0011 (13)	−0.0022 (14)
O13	0.0131 (15)	0.0175 (17)	0.0135 (16)	0.0007 (13)	0.0020 (12)	−0.0006 (13)
O14	0.0195 (18)	0.029 (2)	0.029 (2)	−0.0017 (16)	0.0010 (16)	−0.0053 (17)
O15	0.022 (2)	0.042 (3)	0.025 (2)	0.0091 (18)	−0.0012 (16)	−0.0096 (19)
O16	0.0219 (19)	0.026 (2)	0.032 (2)	−0.0049 (16)	−0.0010 (17)	0.0024 (18)
O17	0.043 (2)	0.028 (2)	0.0164 (19)	0.0016 (18)	0.0006 (17)	0.0017 (16)
O18	0.051 (3)	0.026 (2)	0.030 (2)	−0.017 (2)	−0.010 (2)	0.0036 (19)
O1W	0.0153 (16)	0.0180 (17)	0.0184 (18)	−0.0002 (13)	0.0038 (13)	−0.0010 (14)
O2W	0.0204 (17)	0.0196 (18)	0.0205 (18)	0.0039 (14)	0.0079 (14)	0.0009 (15)
O3W	0.0213 (18)	0.0214 (19)	0.024 (2)	0.0047 (15)	−0.0029 (15)	−0.0008 (16)
N1	0.028 (2)	0.021 (2)	0.016 (2)	−0.0016 (18)	0.0057 (18)	−0.0024 (18)
C1	0.018 (2)	0.017 (2)	0.013 (2)	0.0040 (18)	−0.0013 (18)	−0.0018 (18)
C2	0.020 (2)	0.025 (3)	0.018 (3)	0.001 (2)	−0.0008 (19)	−0.005 (2)
C3	0.017 (2)	0.018 (2)	0.013 (2)	0.0020 (18)	−0.0011 (18)	−0.0007 (18)
C4	0.023 (3)	0.016 (2)	0.032 (3)	−0.001 (2)	0.008 (2)	−0.004 (2)
C5	0.013 (2)	0.019 (2)	0.020 (2)	−0.0014 (18)	−0.0020 (18)	−0.005 (2)
C6	0.025 (3)	0.023 (3)	0.016 (2)	0.007 (2)	0.002 (2)	−0.006 (2)
C7	0.013 (2)	0.022 (2)	0.017 (2)	0.0019 (18)	0.0024 (18)	−0.0037 (19)
C8	0.018 (2)	0.023 (3)	0.024 (3)	0.0000 (19)	−0.001 (2)	−0.003 (2)
C9	0.018 (2)	0.021 (2)	0.010 (2)	−0.0031 (18)	0.0006 (17)	0.0007 (18)
C10	0.020 (2)	0.021 (3)	0.023 (3)	−0.004 (2)	0.001 (2)	−0.002 (2)
C11	0.014 (2)	0.016 (2)	0.016 (2)	−0.0016 (17)	0.0007 (17)	−0.0026 (18)
C12	0.026 (3)	0.027 (3)	0.011 (2)	0.004 (2)	0.0006 (19)	−0.001 (2)
C13	0.019 (2)	0.018 (2)	0.022 (3)	−0.0010 (18)	−0.002 (2)	0.001 (2)
C14	0.024 (3)	0.027 (3)	0.015 (2)	−0.002 (2)	−0.001 (2)	−0.002 (2)

### Geometric parameters (Å, °)

**Table:**

Cr1—O13	1.893 (3)	O18—N1	1.268 (7)
Cr1—O10	1.959 (4)	O1W—H11	0.8400
Cr1—O3	1.962 (4)	O1W—H12	0.8400
Cr1—O1	1.978 (4)	O2W—H21	0.8400
Cr1—O12	2.010 (4)	O2W—H22	0.8400
Cr1—O1W	2.049 (4)	O3W—H31	0.8400
Cr2—O13	1.901 (4)	O3W—H32	0.8400
Cr2—O2	1.969 (4)	C1—C2	1.509 (7)
Cr2—O7	1.970 (4)	C2—H2A	0.9800
Cr2—O5	1.976 (4)	C2—H2B	0.9800
Cr2—O4	1.977 (4)	C2—H2C	0.9800
Cr2—O2W	2.022 (4)	C3—C4	1.506 (7)
Cr3—O13	1.894 (4)	C4—H4A	0.9800
Cr3—O6	1.956 (4)	C4—H4B	0.9800
Cr3—O8	1.978 (4)	C4—H4C	0.9800
Cr3—O11	1.980 (4)	C5—C6	1.511 (7)
Cr3—O9	1.982 (4)	C6—H6A	0.9800
Cr3—O3W	2.075 (4)	C6—H6B	0.9800
O1—C1	1.260 (6)	C6—H6C	0.9800
O2—C1	1.255 (7)	C7—C8	1.508 (7)
O3—C3	1.259 (6)	C8—H8A	0.9800
O4—C3	1.259 (6)	C8—H8B	0.9800
O5—C5	1.281 (7)	C8—H8C	0.9800
O6—C5	1.233 (7)	C9—C10	1.498 (7)
O7—C7	1.272 (6)	C10—H10A	0.9800
O8—C7	1.254 (7)	C10—H10B	0.9800
O9—C9	1.254 (6)	C10—H10C	0.9800
O10—C9	1.267 (7)	C11—C12	1.507 (7)
O11—C11	1.263 (6)	C12—H12A	0.9800
O12—C11	1.269 (6)	C12—H12B	0.9800
O14—C13	1.236 (7)	C12—H12C	0.9800
O15—C13	1.300 (7)	C13—C14	1.492 (7)
O15—H15	0.8400	C14—H14A	0.9800
O16—N1	1.247 (6)	C14—H14B	0.9800
O17—N1	1.228 (6)	C14—H14C	0.9800
			
O13—Cr1—O10	95.17 (16)	Cr3—O3W—H32	109.5
O13—Cr1—O3	98.06 (15)	H31—O3W—H32	109.5
O10—Cr1—O3	166.77 (16)	O17—N1—O16	122.2 (5)
O13—Cr1—O1	93.10 (15)	O17—N1—O18	120.4 (5)
O10—Cr1—O1	87.24 (17)	O16—N1—O18	117.4 (5)
O3—Cr1—O1	92.37 (17)	O2—C1—O1	125.7 (5)
O13—Cr1—O12	93.38 (15)	O2—C1—C2	116.4 (5)
O10—Cr1—O12	92.45 (16)	O1—C1—C2	117.9 (5)
O3—Cr1—O12	86.44 (16)	C1—C2—H2A	109.5
O1—Cr1—O12	173.51 (15)	C1—C2—H2B	109.5
O13—Cr1—O1W	177.86 (16)	H2A—C2—H2B	109.5
O10—Cr1—O1W	82.69 (15)	C1—C2—H2C	109.5
O3—Cr1—O1W	84.09 (15)	H2A—C2—H2C	109.5
O1—Cr1—O1W	86.87 (15)	H2B—C2—H2C	109.5
O12—Cr1—O1W	86.66 (15)	O4—C3—O3	124.2 (5)
O13—Cr2—O2	93.38 (15)	O4—C3—C4	117.7 (5)
O13—Cr2—O7	93.98 (15)	O3—C3—C4	118.0 (5)
O2—Cr2—O7	172.47 (16)	C3—C4—H4A	109.5
O13—Cr2—O5	95.72 (16)	C3—C4—H4B	109.5
O2—Cr2—O5	86.87 (17)	H4A—C4—H4B	109.5
O7—Cr2—O5	90.83 (16)	C3—C4—H4C	109.5
O13—Cr2—O4	94.97 (16)	H4A—C4—H4C	109.5
O2—Cr2—O4	91.88 (17)	H4B—C4—H4C	109.5
O7—Cr2—O4	89.05 (17)	O6—C5—O5	125.8 (5)
O5—Cr2—O4	169.29 (17)	O6—C5—C6	117.9 (5)
O13—Cr2—O2W	178.67 (16)	O5—C5—C6	116.3 (5)
O2—Cr2—O2W	87.02 (16)	C5—C6—H6A	109.5
O7—Cr2—O2W	85.66 (16)	C5—C6—H6B	109.5
O5—Cr2—O2W	85.56 (16)	H6A—C6—H6B	109.5
O4—Cr2—O2W	83.75 (16)	C5—C6—H6C	109.5
O13—Cr3—O6	96.66 (16)	H6A—C6—H6C	109.5
O13—Cr3—O8	94.10 (15)	H6B—C6—H6C	109.5
O6—Cr3—O8	90.55 (16)	O8—C7—O7	125.8 (5)
O13—Cr3—O11	94.52 (15)	O8—C7—C8	117.8 (5)
O6—Cr3—O11	168.79 (16)	O7—C7—C8	116.4 (5)
O8—Cr3—O11	89.43 (16)	C7—C8—H8A	109.5
O13—Cr3—O9	94.02 (15)	C7—C8—H8B	109.5
O6—Cr3—O9	87.33 (16)	H8A—C8—H8B	109.5
O8—Cr3—O9	171.80 (16)	C7—C8—H8C	109.5
O11—Cr3—O9	91.12 (16)	H8A—C8—H8C	109.5
O13—Cr3—O3W	175.13 (16)	H8B—C8—H8C	109.5
O6—Cr3—O3W	88.07 (16)	O9—C9—O10	124.6 (5)
O8—Cr3—O3W	86.97 (16)	O9—C9—C10	118.2 (5)
O11—Cr3—O3W	80.73 (16)	O10—C9—C10	117.2 (4)
O9—Cr3—O3W	85.05 (16)	C9—C10—H10A	109.5
C1—O1—Cr1	129.8 (3)	C9—C10—H10B	109.5
C1—O2—Cr2	133.6 (3)	H10A—C10—H10B	109.5
C3—O3—Cr1	133.1 (3)	C9—C10—H10C	109.5
C3—O4—Cr2	132.6 (3)	H10A—C10—H10C	109.5
C5—O5—Cr2	130.0 (4)	H10B—C10—H10C	109.5
C5—O6—Cr3	134.5 (4)	O11—C11—O12	124.9 (5)
C7—O7—Cr2	133.8 (3)	O11—C11—C12	116.7 (5)
C7—O8—Cr3	130.4 (3)	O12—C11—C12	118.4 (4)
C9—O9—Cr3	128.2 (3)	C11—C12—H12A	109.5
C9—O10—Cr1	133.7 (3)	C11—C12—H12B	109.5
C11—O11—Cr3	135.2 (3)	H12A—C12—H12B	109.5
C11—O12—Cr1	125.8 (3)	C11—C12—H12C	109.5
Cr1—O13—Cr3	119.96 (19)	H12A—C12—H12C	109.5
Cr1—O13—Cr2	119.74 (18)	H12B—C12—H12C	109.5
Cr3—O13—Cr2	120.30 (18)	O14—C13—O15	121.9 (5)
C13—O15—H15	120.0	O14—C13—C14	122.9 (5)
Cr1—O1W—H11	109.5	O15—C13—C14	115.2 (5)
Cr1—O1W—H12	109.5	C13—C14—H14A	109.5
H11—O1W—H12	109.5	C13—C14—H14B	109.5
Cr2—O2W—H21	109.5	H14A—C14—H14B	109.5
Cr2—O2W—H22	109.5	C13—C14—H14C	109.5
H21—O2W—H22	109.5	H14A—C14—H14C	109.5
Cr3—O3W—H31	109.5	H14B—C14—H14C	109.5
			
O13—Cr1—O1—C1	30.3 (5)	O10—Cr1—O12—C11	−49.0 (4)
O10—Cr1—O1—C1	125.3 (5)	O3—Cr1—O12—C11	144.2 (4)
O3—Cr1—O1—C1	−67.9 (5)	O1W—Cr1—O12—C11	−131.5 (4)
O1W—Cr1—O1—C1	−151.8 (5)	O10—Cr1—O13—Cr3	37.4 (2)
O13—Cr2—O2—C1	−6.8 (5)	O3—Cr1—O13—Cr3	−142.2 (2)
O5—Cr2—O2—C1	−102.4 (5)	O1—Cr1—O13—Cr3	124.9 (2)
O4—Cr2—O2—C1	88.3 (5)	O12—Cr1—O13—Cr3	−55.4 (2)
O2W—Cr2—O2—C1	171.9 (5)	O10—Cr1—O13—Cr2	−142.1 (2)
O13—Cr1—O3—C3	−8.3 (5)	O3—Cr1—O13—Cr2	38.2 (2)
O10—Cr1—O3—C3	173.3 (6)	O1—Cr1—O13—Cr2	−54.6 (2)
O1—Cr1—O3—C3	85.2 (5)	O12—Cr1—O13—Cr2	125.1 (2)
O12—Cr1—O3—C3	−101.2 (5)	O6—Cr3—O13—Cr1	−140.3 (2)
O1W—Cr1—O3—C3	171.8 (5)	O8—Cr3—O13—Cr1	128.7 (2)
O13—Cr2—O4—C3	33.4 (5)	O11—Cr3—O13—Cr1	38.9 (2)
O2—Cr2—O4—C3	−60.2 (5)	O9—Cr3—O13—Cr1	−52.5 (2)
O7—Cr2—O4—C3	127.3 (5)	O6—Cr3—O13—Cr2	39.2 (2)
O5—Cr2—O4—C3	−143.3 (8)	O8—Cr3—O13—Cr2	−51.8 (2)
O2W—Cr2—O4—C3	−146.9 (5)	O11—Cr3—O13—Cr2	−141.5 (2)
O13—Cr2—O5—C5	31.9 (4)	O9—Cr3—O13—Cr2	127.0 (2)
O2—Cr2—O5—C5	125.0 (4)	O2—Cr2—O13—Cr1	46.6 (2)
O7—Cr2—O5—C5	−62.2 (4)	O7—Cr2—O13—Cr1	−135.0 (2)
O4—Cr2—O5—C5	−151.5 (8)	O5—Cr2—O13—Cr1	133.8 (2)
O2W—Cr2—O5—C5	−147.8 (4)	O4—Cr2—O13—Cr1	−45.6 (2)
O13—Cr3—O6—C5	−12.1 (5)	O2—Cr2—O13—Cr3	−132.9 (2)
O8—Cr3—O6—C5	82.1 (5)	O7—Cr2—O13—Cr3	45.5 (2)
O11—Cr3—O6—C5	171.9 (7)	O5—Cr2—O13—Cr3	−45.7 (2)
O9—Cr3—O6—C5	−105.9 (5)	O4—Cr2—O13—Cr3	134.9 (2)
O3W—Cr3—O6—C5	169.0 (5)	Cr2—O2—C1—O1	−18.2 (8)
O13—Cr2—O7—C7	−12.7 (5)	Cr2—O2—C1—C2	161.6 (4)
O5—Cr2—O7—C7	83.1 (5)	Cr1—O1—C1—O2	3.0 (8)
O4—Cr2—O7—C7	−107.6 (5)	Cr1—O1—C1—C2	−176.8 (4)
O2W—Cr2—O7—C7	168.6 (5)	Cr2—O4—C3—O3	−9.0 (8)
O13—Cr3—O8—C7	30.7 (5)	Cr2—O4—C3—C4	171.8 (4)
O6—Cr3—O8—C7	−66.0 (5)	Cr1—O3—C3—O4	−6.9 (8)
O11—Cr3—O8—C7	125.2 (5)	Cr1—O3—C3—C4	172.3 (4)
O3W—Cr3—O8—C7	−154.0 (5)	Cr3—O6—C5—O5	−2.5 (8)
O13—Cr3—O9—C9	38.7 (4)	Cr3—O6—C5—C6	177.2 (4)
O6—Cr3—O9—C9	135.2 (4)	Cr2—O5—C5—O6	−10.0 (8)
O11—Cr3—O9—C9	−55.9 (4)	Cr2—O5—C5—C6	170.3 (3)
O3W—Cr3—O9—C9	−136.5 (4)	Cr3—O8—C7—O7	−3.7 (8)
O13—Cr1—O10—C9	6.9 (5)	Cr3—O8—C7—C8	174.9 (3)
O3—Cr1—O10—C9	−174.6 (6)	Cr2—O7—C7—O8	−7.8 (8)
O1—Cr1—O10—C9	−86.0 (5)	Cr2—O7—C7—C8	173.6 (4)
O12—Cr1—O10—C9	100.5 (5)	Cr3—O9—C9—O10	−3.9 (7)
O1W—Cr1—O10—C9	−173.2 (5)	Cr3—O9—C9—C10	174.9 (3)
O13—Cr3—O11—C11	2.2 (5)	Cr1—O10—C9—O9	−25.2 (8)
O6—Cr3—O11—C11	178.1 (7)	Cr1—O10—C9—C10	155.9 (4)
O8—Cr3—O11—C11	−91.9 (5)	Cr3—O11—C11—O12	−13.8 (8)
O9—Cr3—O11—C11	96.3 (5)	Cr3—O11—C11—C12	165.6 (4)
O3W—Cr3—O11—C11	−178.9 (5)	Cr1—O12—C11—O11	−16.4 (7)
O13—Cr1—O12—C11	46.3 (4)	Cr1—O12—C11—C12	164.2 (4)

### Hydrogen-bond geometry (Å, °)

**Table:**

*D*—H···*A*	*D*—H	H···*A*	*D*···*A*	*D*—H···*A*
O1w—H11···O16	0.84	1.96	2.769 (6)	162
O1w—H12···O12^i^	0.84	2.06	2.873 (5)	162
O2w—H21···O14	0.84	1.92	2.668 (6)	147
O2w—H22···O18^ii^	0.84	1.93	2.725 (6)	157
O3w—H31···O14^iii^	0.84	2.28	2.781 (5)	118
O3w—H32···O5^iii^	0.84	2.42	3.244 (6)	165
O15—H15···O18^iv^	0.84	1.81	2.624 (6)	163

Symmetry codes: (i) −*x*+1, −*y*+1, −*z*+1; (ii) −*x*+1, *y*−1/2, −*z*+3/2; (iii) *x*, −*y*+3/2, *z*−1/2; (iv) *x*−1, −*y*+3/2, *z*+1/2.
